# A Retrospective Study of the Epidemiology of Leprosy in Cebu: An Eleven-Year Profile

**DOI:** 10.1371/journal.pntd.0002444

**Published:** 2013-09-19

**Authors:** Pauline F. D. Scheelbeek, Marivic V. F. Balagon, Florenda M. Orcullo, Armi A. Maghanoy, Junie Abellana, Paul R. Saunderson

**Affiliations:** 1 Department of Epidemiology and Biostatistics, Imperial College London, London, United Kingdom; 2 Epidemiology - Clinical, Leonard Wood Memorial Centre for Leprosy Research, Cebu, Philippines; 3 American Leprosy Mission, Greenville, South Carolina, United States of America; Fondation raoul Follereau, France

## Abstract

**Background:**

Cebu has been one of the most leprosy endemic areas in the Philippines. Despite the high coverage rates of multiple drug therapy (MDT) and high BCG-vaccine coverage in children, leprosy control authorities believe that leprosy transmission and incidence (as evidence by continuing new case detection in both adults and children) have not declined as expected, once leprosy had been eliminated.

In response to the concerns communicated by the authorities regarding ongoing leprosy transmission in Cebu, this study aims to examine the evidence for the hypothesized ongoing transmission, both in children and adults. Furthermore, it will be assessed which groups and areas are experiencing a continuing risk of leprosy infection; this can form a starting point for more targeted approaches to leprosy control.

**Methodology & Principal Findings:**

Case records from 2000–2010 were retrospectively collected from the Leonard Wood Memorial Clinic archives, and all other clinics on the island where leprosy was treated. Between 2000 and 2010, 3288 leprosy cases were detected. The overall five year case notification rate (CNR) dropped significantly from 47.35 (2001–2005) to 29.21 cases (2006–2010) per 100.000 population. Smaller CNRs were reported for children; however the decline in child-CNR over the same period was minimal. Furthermore, no increase in median age of notification in children or adults was found between 2000 and 2010. Population-adjusted clustering of leprosy cases was mainly detected in urban and peri-urban areas.

**Conclusions & Significance:**

Although the overall CNR declined significantly, CNR seems to be rather static in lower risk populations and areas. Cases are mainly found in urban areas, however CNRs in these areas decline at a much faster rate than in the lower endemic rural areas. A similar situation was found when comparing adults and children: CNRs observed in children were lower than in adults, but further decline (and elimination) of these childhood CNRs was found to be difficult. Moreover, the median age of notification in children has remained stable, suggesting transmission is still on-going.

It is unclear why many years of good MDT-coverage and a gradual decline in CNR have not been accompanied by evidence of reduced transmission, especially beyond a certain threshold level of case notification. We believe that a new approach to leprosy control is required to tackle transmission more directly. The most promising approach may involve chemoprophylaxis and/or immunoprophylaxis interventions, targeted at high risk (urban) areas and groups such as household contacts, followed by a different approach once decline in CNR starts to level off. Identified clusters and trends can form the starting point for implementing this approach.

## Author Summary

The island of Cebu is one of the most leprosy-endemic areas in the Philippines. Multiple drug therapy (MDT), improved BCG-vaccine coverage and active case finding have significantly lowered the adult case notification rates (CNRs), but the CNR in children (which is a proxy indicator of ongoing transmission) seems to be more static over the last decade (2000–2010). The long incubation period of leprosy, hampers determination of time of infection, however one would expect the median age of notification in children to increase when transmission decreases, as younger subjects would have a lower risk of infection: in this study no significant changes in median age were found between 2000–2010. Furthermore, leprosy seems to be mainly confined to urban areas, where nonetheless the measured decrease in CNR is much larger than in less endemic rural areas. It is unclear why the significant decline in CNR have not been accompanied by evidence of reduced transmission and why CNR seem to level off beyond a certain threshold level. We believe that more targeted approaches (e.g. focused on household contacts in urban areas) involving chemoprophylaxis and/or immunoprophylaxis, followed by a specific approach for lower CNRs, are required to tackle leprosy more directly.

## Introduction

For over a century, Cebu has been one of the most leprosy endemic areas in the Philippines. With the establishment of a centralized leprosy settlement in the island of Culion, compulsory notification of leprosy cases was introduced in 1906; ever since, a large proportion of leprosy cases have been coming from Cebu. Because of the high endemicity, another leprosy center was established in Cebu in 1930, through the Leonard Wood Memorial Leprosy Research Center (LWM). Over the next two decades, LWM conducted a number of classic epidemiological leprosy studies in the area, particularly in the municipality of Cordova. Much of our basic knowledge of the epidemiology of leprosy comes from these early population surveys [1], [2].

To reduce the global burden of disease associated with leprosy, the World Health Organization introduced Multiple Drug Therapy (WHO-MDT) in 1982. WHO-MDT is a convenient, relatively inexpensive regimen consisting of monthly rifampicin and clofazimine, and daily dapsone and clofazimine administered for 2 years (more recently, this was reduced to one year) in multibacillary leprosy. By 1994, MDT was implemented worldwide and the overall prevalence of leprosy dropped dramatically [3]. In 1985, MDT was implemented in the Philippines. After a few years, relatively good coverage rates were observed particularly in the island of Cebu [4], [5] . The LWM clinic, which handles the majority of Cebu leprosy cases, documents through its internal reports an MDT completion rate of >95% among its diagnosed cases since 1990 [6]. The fall in global prevalence with the introduction of MDT led to the WHO campaign to eliminate leprosy as a public health problem by the year 2000, with the assumption that once prevalence fell below the target figure of 1 case per 10^5^, transmission would be interrupted, leading to the gradual extinction of the disease [7]. If this process were in fact happening, health authorities would be justified in reducing the allocation of resources to leprosy control.

In addition, BCG vaccination, which is believed to have some prophylactic effect against both tuberculosis and leprosy [8], has seen increasing coverage in the past 30 years; from less than 70% in the 1980's to over 90% in the 2000's [9].Currently the BCG coverage is estimated at 88% throughout the Philippines [9] , including the island of Cebu.

Despite these measures, however, studies in the last decades have shown that the expected decline in the new case notification rate (CNR) and possibly incidence of leprosy has not occurred [3], [10]–[14]; in some high endemic areas even an increase in leprosy notification rates is found [10], [15]–[17]. Globally, approximately 250,000 new leprosy cases are still detected each year [17].

It is of crucial importance that the concerns of health authorities on possible ongoing transmission are studied and high risk groups and areas will be identified. Therefore, in this study we will examine whether transmission is continuing in Cebu, examining overall and group-specific CNRs and trends over 11 consecutive years (2000–2010), as well as spatial and spatio-temporal trends. Special attention will be paid to childhood leprosy.

The province of Cebu (consisting of the island of Cebu and a few smaller islands close by) is sub-divided into 53 municipalities, of which four comprise the greater conurbation of Cebu City and its suburbs (these are Cebu City, Lapu-Lapu City, Talisay and Mandaue City). Approximately 40% of the island's population of 4.2 million lives in this conurbation. LWM's Skin Clinic is in Cebu City, while the residential leprosy center established in 1930 is in Mandaue City; Cordova is adjacent to Lapu-Lapu City on nearby Mactan Island.

Analyzing and interpreting more than a decade's worth of data will enable us to better understand possible transmission patterns and risk profiles and will help us to prioritize and optimize leprosy control strategies.

## Materials and Methods

Clinical and demographic information of leprosy cases, detected between 2000 and 2010 through standardized questionnaires, was retrospectively collected from clinical records archived at the LWM clinic and other leprosy treatment facilities throughout the island.

All patients had been diagnosed in the same way: first they were checked for the presence of skin and/or nerve lesions consistent with leprosy. Then, slit skin smear examination was performed to demonstrate the presence of acid-fast bacilli. Subsequently patients were further classified into multi-bacillary (MB) or pauci bacillary (PB) patients, using the WHO Classification based on lesion and bacterial count. MB was defined as smear positive (with any number of lesions) or smear negative with more than five lesions, while the PB-classification was assigned to smear negative patients that had a maximum of five lesions. All patients diagnosed in LWM were also histologically classified using the Ridley-Jopling Scale.

At the time of diagnosis a standardized questionnaire was used to collect information on home location, sex and age of the patients. This form was also used to store information on date of notification and leprosy type. Whenever necessary, uncertainties in the records were validated through house visits in the weeks after the diagnosis.

Data gathered from the archives was encoded and collated for spatial and statistical analyses as well as Geographic Information Systems (GIS) mapping. The period of analysis was restricted to 2000–2010 because of the similar approach of case notification during those years; active case finding activities were carried out in both 1999 and 2011, which therefore show considerably higher rates than the period under study.

CNR was defined as the total number of cases detected per 100.000 population. Census data from 2000, 2007 and 2010 were used, and linear interpolations were made for the years in between the censi. The overall and stratified trends in CNR and CNR-ratios were calculated using autoregressive integrated moving average (ARIMA) models. This type of model allowed accounting for time-dependent disturbances (such as unknown delays in diagnosis), and possible clustered clinic visits (for example, if someone was diagnosed in the area). Sub-group analysis was performed based on leprosy type, sex, age and area of residence. Data were checked for linearity and residuals were analysed on skewness and kurtosis. Furthermore stationary P-values were obtained. With this information, the best fit ARIMA model was selected based on the Akaiki's Information Criterion (AIC) and Ljung-Box Q values.

For comparison of median age of leprosy in children, the Mann-Whitney U test was used. Median age was adjusted for leprosy type. All calculations were performed in Stata version 12.0.

Spatial clusters were detected using area-based data aggregated to municipality level. A Poisson-based model was used; this model is based on a Poisson distributed number of events (leprosy cases) according to a known underlying population at risk [18]. Cebu City, Lapu-Lapu City, Talisay and Mandaue City were considered as a separate clusters. Further analysis on a lower scale within these cities was not possible due to small case numbers and the restricted time period. Mapping and spatial analyses were performed in R-2.9.0 and ArcGIS 10.1.

### Ethics statement

With the approval of LWM's Institutional Ethical Review Board, the study was conducted in coordination with the Regional Health Authorities and all leprosy treatment facilities throughout the island. All data used were analyzed anonymously.

The abstracted register data was digitalized by a trained database manager and anonymized before sharing the data with the data analyst/statistician. In the rare occasion a household visit had to be conducted, oral consent was obtained from the patient. This was selected as the preferred method of obtaining consent (above written consent) in view of the lower literacy rates in the rural areas of Cebu Island; oral consent would minimize embarrassment of the patient related to literacy. The Institutional Ethical Review Board approved this decision. There were no patients that refused consent in this study.

## Results

### I. Total cases and overall notification rates

Between 2000 and 2010, Cebu detected a total of 3288 leprosy cases (Table 1). A significantly declining case notification was found over the selected 11-year period; from 319 cases (CNR: 12.0 per 10^5^ population) notified in 2000 to 204 cases (CNR: 4.8 per 10^5^ population) in 2010. The ARIMA (1.1.0) model shows an annual decline of 0.715 [0.474/0.955, P = 0.002]] cases per 10^5^ population per year (first order Autoregressive Correlation Coefficient (ACC) −0.820 [−1.192/−0.448]; Ljung-Box χ^2^: P = 0.8592).

**Table 1:** Leprosy cases and case notification rates in Cebu (by sex).

Year	Total Cases	MB cases (%)	Male Cases	Female Cases	Total CNR	Male CNR*	Female CNR*
	(N = 3288)	(N = 2770)	(N = 2281)	(N = 1007)	Per 10^5^	Per 10^5^	Per 10^5^
2000	391	316 (80.8%)	260	131	11.99	15.95	8.04
2001	356	280 (78.7%)	227	129	10.36	13.21	7.51
2002	363	301 (82.9%)	274	89	10.31	15.56	5.05
2003	337	264 (78.3%)	210	127	9.34	11.64	7.04
2004	370	314 (84.9%)	254	116	10.01	13.74	6.28
2005	283	259 (91.5%)	193	90	7.48	10.20	4.76
2006	289	245 (84.7%)	210	79	7.46	10.84	4.08
2007	236	198 (83.9%)	169	67	5.95	8.53	3.38
2008	263	236 (89.7%)	193	70	6.46	9.48	3.44
2009	196	177 (90.3%)	141	55	4.70	6.77	2.64
2010	204	180 (88.2%)	150	54	4.79	7.05	2.54
	***Trend/***		***yr:***		***−0.715***	***−0.887***	***−0.558***

* Male and female populations estimated on 50% of total population.

When splitting up the rural/per-urban municipalities from the four large urban areas, the data shows a drop in CNR in rural areas from 7.50 cases per 10^5^ population in 2000 to 3.50 cases per 10^5^ in 2010. In urban areas these numbers are 18.43 and 6.67 respectively. Using an ARIMA (1,1,0) model an annual decrease in CNR of 0.380 [0.144/0.617 P = 0.002] cases per 10^5^ population in the rural/peri-urban municipalities can be observed (first order ACC: −0.753 [−1.362/−0.144]; Ljung-Box χ^2^: P = 0.8871). The four larger cities, Cebu-City, Lapu-Lapu City, Mandaue City and Talisay showed in a similar model a much higher annual decrease of 1.19 [0.389/1.986, P-value = 0.004] cases per 10^5^ population (first order ACC: −0.580, [−1.346/0.185], Ljung-Box χ^2^: P = 0.9509). It appears that in urban areas the CNRs are higher, but decrease more rapidly than in the lower endemic rural areas.

When looking at percentagewise differences in CNR over two periods (Period 1 = 2001–2005, Period 2 = 2006–2010), we learn that the overall CNR reduces by 27.8% over period 1, and 35.7% over period 2. In the rural/peri-urban municipalities these percentages are 30.0 and 30.4 respectively and for the four larger cities 29.4% and 38.6%. The home address from 52 patients was missing.

CNRs for MB and PB cases were separately analyzed. Overall the MB-CNR reduced from 9.45 cases per 10^5^ population in 2000 to 4.23 cases per 10^5^ population in 2010. Using an ARIMA (1,1,0) model, an annual reduction of 0.509 [0.144/0.873] cases per 10^5^ population can be observed (first order ACC = −0.810 [−1.519/−0.100], Ljung-Box χ^2^:P = 0.9262). The PB-CNR reduced from 2.23 cases per 10^5^ in 2000 to 0.56 cases per 10^5^ in 2010. An ARIMA (2,1,0) model was selected and showed an annual decrease of 0.180 (0.056/0.305) cases per 10^5^ population (no significant 1^st^ and 2^nd^ order ACCs; Ljung-Box χ^2^: P = 0.661 & 0.881).

Overall MB-CNR showed a 55.2% reduction over 11 years (18.0%in period 1 and 32.6% in period 2), while PB showed a 74.8% reduction over eleven years (73.0% and 49.2%, respectively). The ARIMA (0,1,1) model detected a small significant increase in the proportion of patients who were MB of 1.05% [0 .22–1.87%] per year.

### II. Detecting target groups

#### a. Case notification rates by sex

Significant downwards trends were found for both men and women when stratifying CNRs for sex: an annual decrease of 0.887 and 0.558 (ARIMA (1,1,1) cases per 10^5^ population per year was found for the male and female populations, respectively (Table 1).

The male-female ratio increases gradually over the 11 measured years; the trend is however not significant. Although CNRs in men are higher than in women, proportional changes over time are similar, therefore there is no evidence that relative risks for men and women have changed significantly over the past 11 years.

#### b. Case notification rate by age

**Figure 1:**
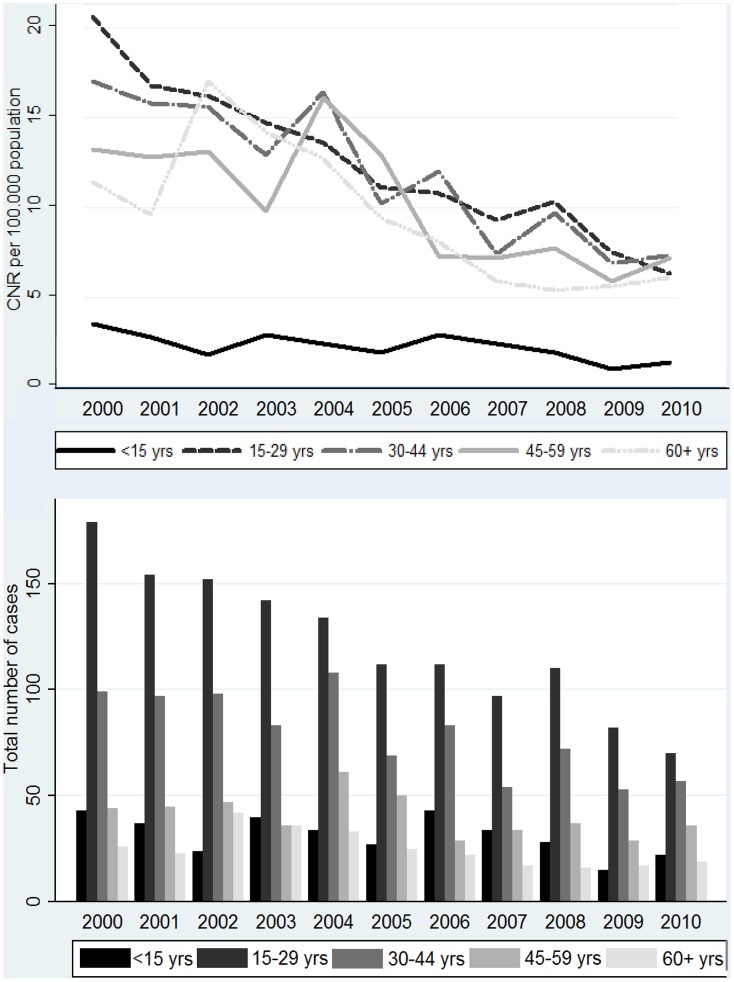
Leprosy cases (bar graph) and case notification rates (line graph) by age group.

Stratified analysis (Figure 1) showed declining trends in all age groups. For each age group a significant decrease in CNR was observed (ARIMA (0,1,1) model). The highest decline was found among the 15–29 year olds (−1.27 cases per 10^5^ per year, [−1.49/−10.6], P = <0.001) and the least decline in the youngest age group (−0.16 cases per 10^5^ per year, [−0.30/−0.03], P = 0.020).

When comparing five-year CNRs among adults (15+) and children (<15), we see a considerable decrease in CNR among adults from 69.37 cases per 10^5^ population over five years in the first period to 40.60 in the second period. The decrease in children is much smaller from 11.75 to 9.52 cases per 10^5^ population over five years. However over the 11 year period, the percentagewise decrease is similar in adults and children. This was 60% in children, and 69, 51, 45 and 46% respectively in the subsequent age groups. In section III this group will be further explored.

The percentage of child cases remains stable in the period under study: from 11.0% in 2000 to 10.8% in 2010 (range 6.6–14.9).

### III. Children <15 years of age: A closer look

#### a. Overall case notification rates in children <15 years

Pediatric cases are mainly reported in the four large urban areas. In some rural areas the case numbers were low, therefore data was analyzed in 5-year periods, rather than analysis of annual trend: the numbers for individual rural municipalities were too small to identify clusters and/or trends. In Table 2, the cases over a five-year period are shown, as well as the CNR per 10^5^ children <15. We see a decrease in CNR in all 4 cities comparing period 2 with period 1. In the combined analysis of rural areas, we can see that the CNR in children shows a slight increase, rather than decrease when comparing period 1 with period 2. The CNR values in these areas, are however lower than in urban areas throughout the study period. The MB/PB ratio changes differently in each area.

**Table 2:** Leprosy case notification rate in children (per 100000) in four urban areas with endemic leprosy and all rural areas combined.

		Cebu City		Lapu-Lapu City		Mandaue City		Talisay		Rural/peri-urban areas	
		Cases	CNR	Cases	CNR	Cases	CNR	Cases	CNR	Cases	CNR
2001–2005	*Total*	30	12.6	22	28.0	30	32.9	10	14.5	61	7.7
(period 1)	*MB*	19	8.0	16	20.3	24	26.3	4	5.8	44	5.5
	*PB*	11	4.6	6	7.7	6	6.6	6	8.7	17	2.1
	*MB/PB ratio*	*1.73*		*2.67*		*4.00*		*0.67*		2.59	
2006–2010	*Total*	19	7.7	19	18.0	25	25.7	5	6.8	72	8.5
(period 2)	*MB*	13	5.3	16	15.2	16	16.4	5	6.8	57	6.7
	*PB*	6	2.4	3	2.6	9	9.2	0	0	15	1.8
	*MB/PB ratio*	*2.16*		*5.33*		*1.78*		*-*		3.80	
**CNR-ratio** *	*Total*		**1.64**		**1.56**		**1.28**		**2.13**		**0.91**
**p1 versus p2**	*MB*		**1.51**		**1.34**		**1.60**		**0.85**		**0.83**
	*PB*		**1.92**		**2.96**		**0.72**				**1.22**

* As a comparison adult CNR-ratios (period 1 vs. period 2) for Cebu City, LL City, Mandaue City, Talisay and rural areas were 1.97, 1.90, 1.62, 1.67 and 1.58 respectively.

#### b. Median age (and interquartile range) of notification in children <15

Of the 3288 cases being reported, 407 (12.4%) were aged under 15 years. Of these 24 (6%) were under 5 years old, 158 (39%) were aged 5–9 years, and 225 (55%) were aged 10–14.

**Figure 2:**
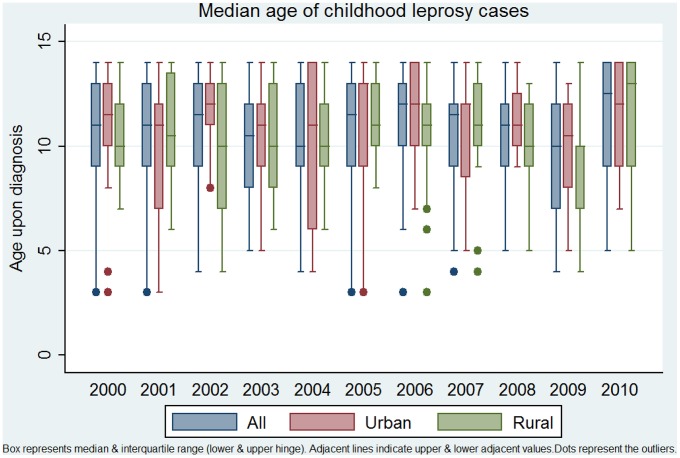
Median age upon diagnosis- total and urban/rural (2000–2011).

In Figure 2, the median ages of all pediatric leprosy cases are displayed, stratified for urban and rural municipality, and MB/PB status. No difference in median was found for urban versus rural child cases (Mann Whitney U, P-value = 0.1734), nor when comparing period 1 (2001/2005) with period 2 (2006–2010, P-value 0.5553).

A difference was observed for MB and PB patients, with a median age of 11 (9–13) and 10 (8–12) respectively. When comparing period 1 with period 2 for MB and PB child cases separately, no change in median age was found (Mann Whitney U test P-values 0.7387 and 0.7979) for MB and PB cases separately.

The MB/PB ratio in children slightly changed from 69.8% MB cases in period 1 to 76.6% in period 2. Following an ARIMA (1,1,0) model, the change in MB/PB ratio was however not significant (P = 0.793).

As a comparison, trends in median age of diagnosis for all patients were assessed (children and adults combined): No significant decrease or increase could be detected, even when analyzing MB and PB patients separately.

### IV. Detecting risk areas

#### a. CNR per city/municipality

**Figure 3:**
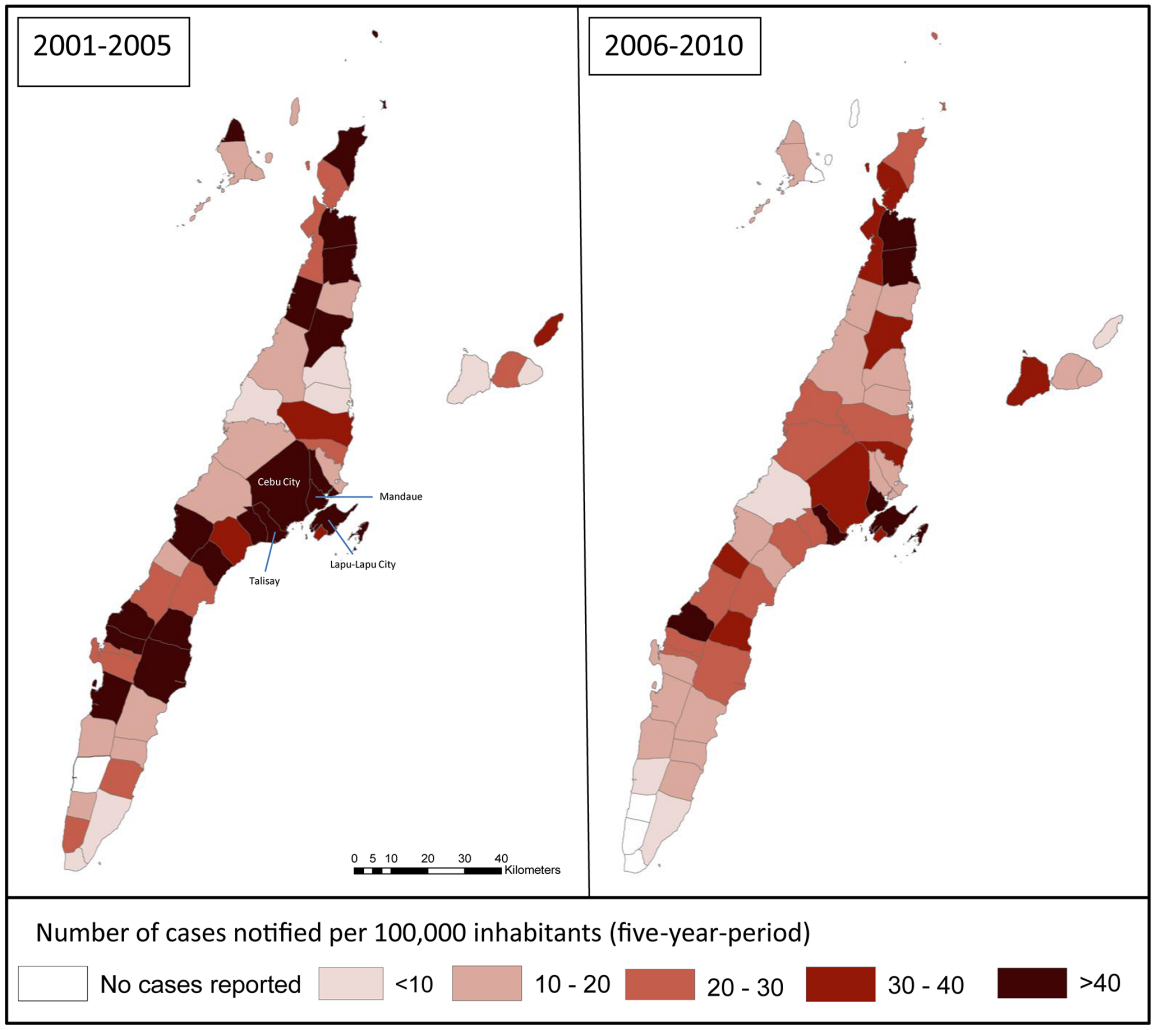
Leprosy case notification rates per municipality/city per 100000 populations.

**Figure 4:**
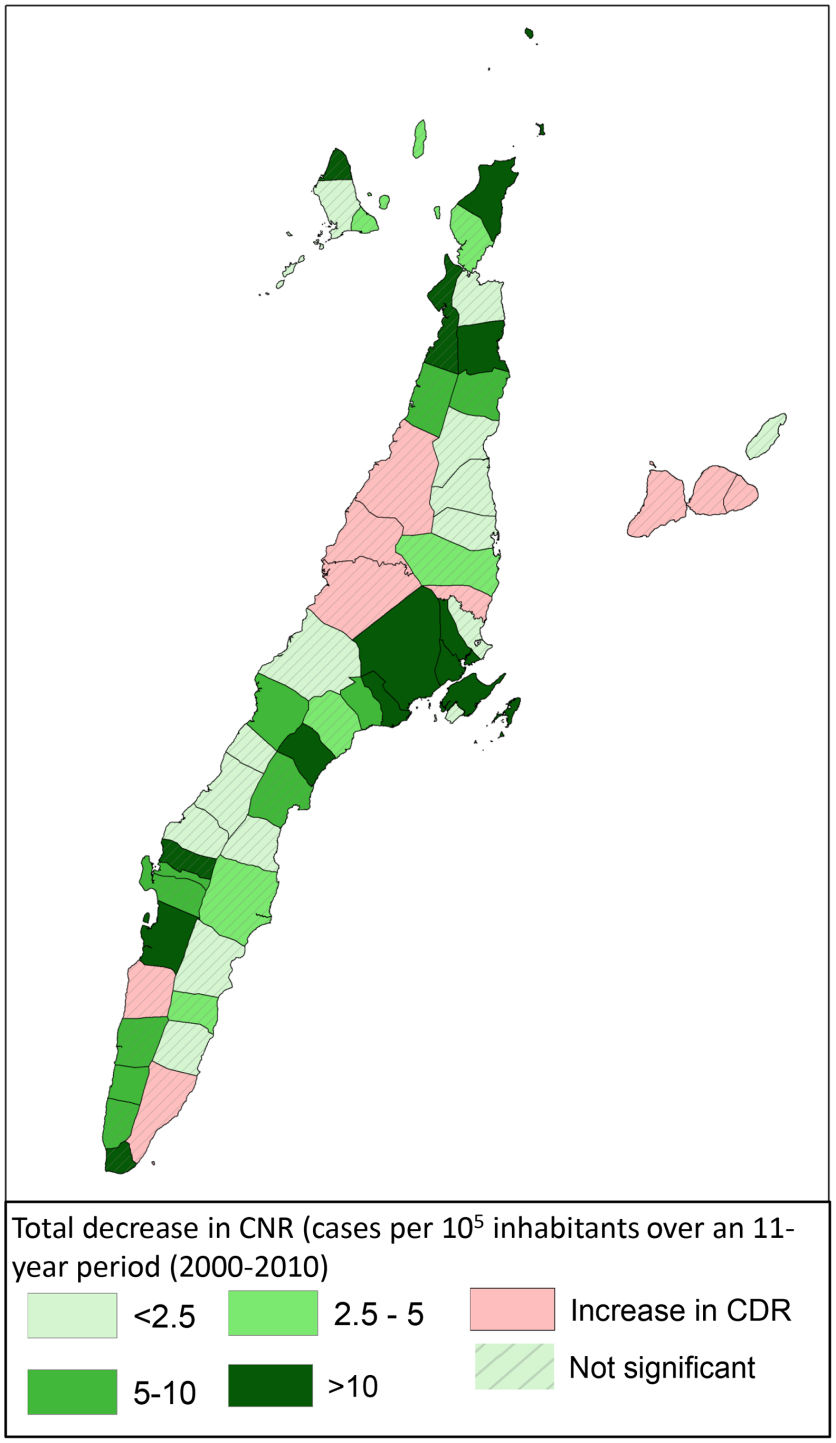
Changes in leprosy CNR over a 5 year period (2001–2005 versus 2006–2010).

In Figure 3, an overview of CNR per 10^5^ population is presented for each of the 53 municipalities/cities in Cebu. Using an first order ARIMA model, we see a decline of CNR over the study period in 44 of the 53 municipalities/cities; this trend is significant in 12 municipalities/cities. In the remaining 9 municipalities/cities we see a non-significant increase in CNR. Figure 4 shows the modeled changes in CNR per municipality/city over an 11-year period; the non-significant results are hatched.

#### b. CNR in children per city/municipality

**Figure 5:**
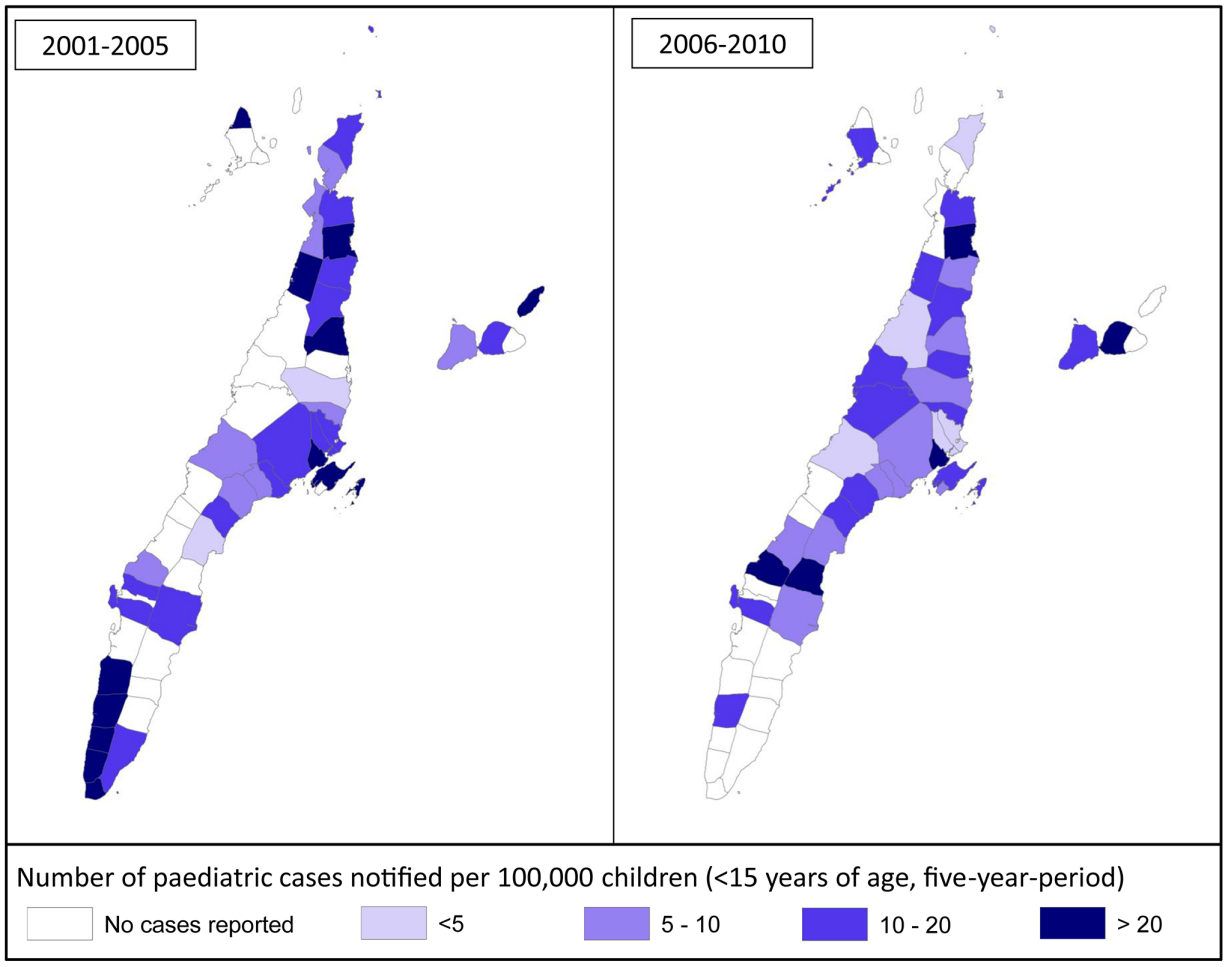
Leprosy CNR in children (per 1000000) per municipality/city in Cebu.

We investigated whether a larger (significant) decrease of CNR in a certain area would show a similar reduction in child CNR in that same area. Figure 5 presents an overview of child CNR over 5 year periods (2001–2005/2006–2010). Although some patterns can be observed, the association between changes in overall CNR per municipality/city and changes in child CNR was not significant, possibly partly due to small case numbers in some municipalities.

#### c. Population adjusted spatial clustering of cases

**Figure 6:**
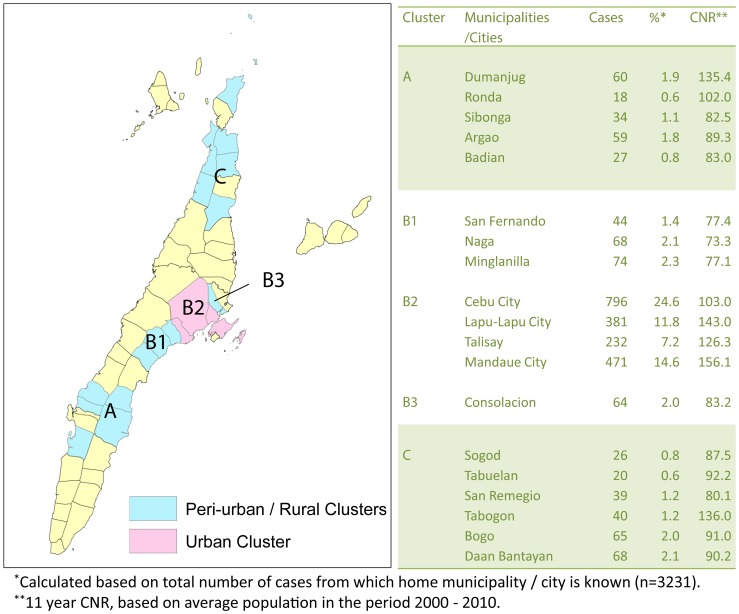
Statistically significant clustering of leprosy cases over an 11-year period (2000–2011).

In all 4 previously described urban areas (Cebu City, Lapu-Lapu City, Mandaue City and Talisay) CNR is significantly higher over an 11 year period than in the surrounding municipalities. Cluster analysis was performed separately for the remaining (peri) urban and rural municipalities. The results are presented in Figure 6. Significant clusters are highlighted.

**Figure 7:**
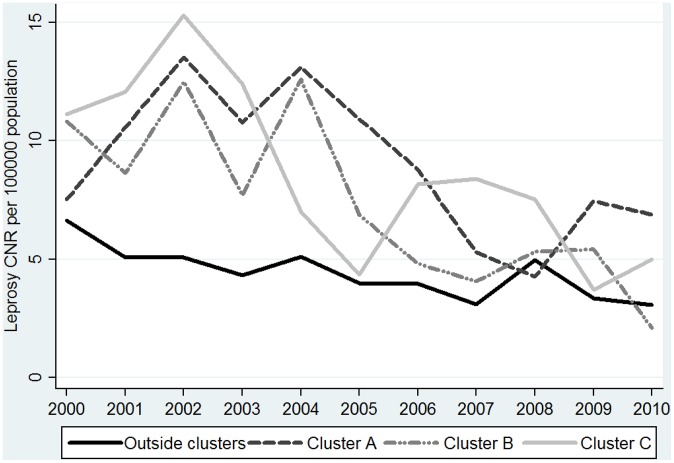
Annual CNR for rural/peri-urban municipalities within and outside leprosy clusters.

When comparing the changes in CNR of municipalities outside the cluster with those within, we see that the CNR from municipalities in the cluster areas (with higher case numbers and CNRs) declines more rapidly than in the municipalities outside the cluster area. The latter show in an ARIMA (0,1,1) model a (combined) annual decrease of 0.26 [0.08–0.88 cases per 10^5^ population, while the municipalities in cluster A, B and C show higher annual decreases of 0.47 [−0.31/1.25]; 0.86 [0.36–1.37] and 0.79 [0.11–1.46] cases per 10^5^ population respectively (ARIMA (0,1,2 model). This apparent effect can be observed when plotting the annual CNR- values in a graph (see Figure 7).

## Discussion

Leprosy has been studied intensively in Cebu for several decades, beginning with population surveys in one municipality conducted by Doull et al. [1], [2] during the 1930s and 40s. Further studies, including clinical trials of treatment regimens, have been conducted more recently, suggesting that basic leprosy control activities have generally been well supervised in the island. It is often assumed that good case-finding and chemotherapy with MDT, as well as a background of good coverage with BCG immunization in infants, would lead to a diminution of leprosy transmission and a decline in the incidence of leprosy. In this study, we have studied this hypothesis for Cebu Island, Philippines. We have used case notification as a proxy for incidence, taking care to ensure that case notification methods remained the same during the study period, and median age of diagnosis in children as proxy for ongoing transmission.

It is an enigma that despite good MDT coverage for many years and a gradual decline in CNR, the transmission of leprosy appears to be continuing in Cebu. Furthermore, the recent analysis of data from many countries has shown that the global decline in leprosy case detection has been less than expected, despite widespread use of MDT [3]. Actual numbers of new cases reported for the last 5 years are very stable, while detection rates decline slowly due to rising population figures [9].

Our results suggest that the decline in CNR over the study period is often higher in sub-groups and areas with a higher ‘start CNR’, and seems to decline very slowly if the CNR in 2000 was already relatively low. This could indicate a threshold, after which it becomes more difficult to lower CNR. This observation has been very consistent throughout our results; the decline of CNR was much faster for high endemic urban areas than in lower endemic rural areas (and those outside clustered leprosy areas), for men (high CNR) it declined much faster than for women (lower CNR), and for adults (in different age groups) the decline was much faster than in children (with lower CNRs). This suggestion is however not in line with the reduction in CNR of PB versus MB patients, where we observed that the lower CNR of PB-cases drops slightly faster than the CNR of MB patients. This could be a result of the fact that only passive case finding was conducted in the period under study, following active case finding activities in 1999.

During the decade 2000–2010, a total of 3288 leprosy cases were detected in the island of Cebu, with a declining trend. Despite these promising reductions in overall CNR over the last 11 years, notification rates in children are declining much slower, suggesting transmission is still ongoing. The CNR in children under 15 years of age has remained quite stable at around 2 per 100,000 population (Figure 1) and the median age of children at diagnosis (approximately 11 years) has not changed significantly over the decade Figure 2. If transmission had been greatly reduced over the last two decades, as many believed would occur as a result of the elimination campaign, one may expect a reduced number of child cases, especially in the youngest age group and rising average or median age at diagnosis, amongst those children who do get leprosy. It should be noted that the MB/PB ratio should be taken into account, when comparing median age of diagnosis, as a general tendency of earlier/later diagnosis (as result of awareness campaigns, changes in health care fees etc.) could distort age analysis based on CNR.

In his comprehensive review, in 1985, of age-specific leprosy data in situations of gradually declining incidence, Irgens [14] demonstrates that in both leprosy and tuberculosis, two apparently opposing trends can be identified as the overall incidence declines, which may make interpretation more complicated. The evidence suggests that when transmission still occurs in a population, infection tends to occur at quite a young age, but because of the variable and often very long incubation period, the onset of disease may be at any age. Thus, on the one hand, in cross-sectional studies (looking at all the new cases in any particular year), older people will have been infected at a time of higher rates of transmission, when they were children, and will therefore have a higher lifetime risk of developing disease; they will be over-represented amongst new cases, so the age of onset of active disease will appear to be gradually increasing, as less and less disease is diagnosed in young people who have a lower lifetime risk of disease. On the other hand, if one examines disease in any particular cohort (for example, everyone born in 1950, or 1999, etc.), a different pattern will be observed, with a maximum incidence at age 15–25 years; the overall incidence in the later cohorts will be less, but the general pattern will remain the same for each cohort examined [14].

In Irgens' editorial [14], it is worth noting in greater detail the reported trend of CNR in children in Norway, between 1851 and 1920 (his figure 1), which declined from around 15 per 10^5^ to 0.1 per 10^5^ population. The rate declines to just under 2 per 10^5^ population in the period 1881–1900, and then to 0.1 per 10^5^ for the period 1901–1920. The periods of review in our study are shorter (two 5 year periods), but it seems surprising that only a very small reduction in childhood CNRs was detected, if leprosy really is being eliminated

Surprisingly, although the prevalence rate of leprosy was much higher in the 1930s, Doull et al [1] reported that childhood leprosy occurred in the different sub-groups in very similar proportions to those reported here: 3 (5%) in the age-group <5 years; 22 (38%) aged 5–9; 33 (57%) aged 10–14, amongst a total of 58 cases under 15 years of age; in the current study the proportions are 6%, 39% and 55% respectively. In the COLEP study in northern Bangladesh [19], over the first two years of follow-up, under 5s were excluded from the study, but there were 5 (29%) incident cases aged 5–9, and 12 (71%) aged 10–14. Most studies do not break down the <15 age group into smaller sub-groups, so it is difficult to speculate further on the significance of these findings. The close similarity in the proportions of children of different ages affected now, as compared with the proportions in the 1930s, suggest that some aspects of leprosy transmission to the next generation may have changed less than we think.

The data also show, however, that in specific areas where the greatest reduction in overall CNR has taken place, particularly in the Cebu City area, this seems to be associated with a lowering child CNR (although not significant in this study). One possible explanation may be rising living standards in the urban metropolis of Cebu City which may have a greater effect on transmission than other measures. The corollary of this is that in the other parts of the island, transmission of leprosy to the next generation appears to be continuing unchecked. Children continue to represent slightly over 12% of cases, a long way from WHO's goal of <3%, suggesting continuing transmission of leprosy in the island.

Another possibility in relation to continuing transmission is the development of drug resistant leprosy. Dapsone resistance developed in many places in the 1960s and 70s, leading to the introduction of MDT in 1982. Since that time, drug resistance has not been a problem and in recent years this has been confirmed by a drug resistance surveillance program set up by WHO in 2006 [20].LWM is the sentinel site in the Philippines for surveillance of drug resistance in leprosy and has contributed to the development of field-friendly tests for rifampicin resistance [21]. These studies have failed to show any rifampicin resistance in leprosy in the Philippines at the present time.

This study was subject to a number of limitations. First of all, the study is retrospective and no real-time verification could be made. The study uses data from a period of only passive case finding: the CNRs might therefore not be representative for incidence, and trends might have been over or under estimated. The Island of Cebu is however relatively well covered by leprosy clinics and many satellite clinics, making it accessible for the Cebu population, minimizing the gap between CNR and real-time incidence. The case numbers in some areas are small and this study evaluates a relatively short period, conclusions might therefore be somewhat tentative. However, despite these limitations, the Cebu community is stable and of manageable size; therefore the data is believed to be relatively reliable. LWM works in close collaboration with government clinics and health authorities, which suggests good reliability of the data.

In conclusion, our study shows that leprosy transmission is still very active in the island of Cebu, despite good coverage with MDT and BCG in recent decades. It seems that especially in groups and areas with lower leprosy rates, such as children and people in rural areas, further reduction of CNR (and eventually elimination) is difficult to establish. We believe that a new approach to leprosy control is required to tackle the issue of transmission more directly; the most promising approach is likely to involve interventions, such as chemoprophylaxis and/or immunoprophylaxis, targeted at high risk groups, such as household contacts and high risk areas, with a subsequent specific approach once declines in CNRs start to level off. In Cebu, the cluster analysis in Figure 6 (looking at both trends in space and over time) could be used to further target new interventions.

## Supporting Information

Checklist S1 STROBE checklist.
(DOC)
